# Phylogenetic relationship of Chinese pangolin (*Manis pentadactyla aurita*) revealed by complete mitochondrial genome

**DOI:** 10.1080/23802359.2020.1772693

**Published:** 2020-06-22

**Authors:** Yan Hua, Jiao Wang, Fuyu An, Jinqian Xu, Han Zhang, Hexiang Gu

**Affiliations:** a Guangdong Provincial Key Laboratory of Silviculture, Protection and Utilization, Guangdong Academy of Forestry, Guangzhou, China; b College of Wildlife and Protected Areas, Northeast Forestry University, Harbin, China; c Guangdong Provincial Wildlife Rescue Center, Guangzhou, China

**Keywords:** mtDNA genome, phylogenetic relationship, Chinese pangolin, *Manis pentadactyla*

## Abstract

The Chinese pangolin (*Manis pentadactyla*) is an extremely endangered species, it has been banned from international trade due to a sharp decline of the population number in China. It is difficult to distinguish among subspecies, thus making it entangled in law enforement. In order to clarify this chaos, we determined and annotated the whole mtDNA genome of the Chinese pangolin. The complete mitogenome is 16 573 bp in length, includeing 13 protein-coding genes, 22 tRNA genes, 2 rRNA genes, and one control region. We built the phylogenetic tree of Chinese pangolinand other 7 most related *Manis* species.

Due to loss of habitat, excessive consumption of traditional Chinese medicine, indiscriminate poaching and other reasons, the distribution and population of Chinese pangolins in China has declined sharply (Li et al. 2018). It is estimated that the population of the Chinese pangolin has fallen by about 94% (Wu et al. 2004). In 2014, the Chinese pangolin was listed as a critically endangered species (CR) by the International Union for Conservation of Nature (Challender et al. 2019). In 2015, the Chinese Red List of Biodiversity also evaluated pangolins as ‘extremely endangered’. In 2017, it was listed as an Appendix I species by CITES, and it is prohibited for international trade.

There are three subspecies of Chinese pangolin, including *Manis pentadactyla pendactyla* (Khatri-Chhetri et al. 2015), *Manis pentadactyla pusilla* (Helen et al. 2016) and *Manis pentadactyla aurita* (Jiang 2015; Yu & Peng 2016; Fan et al. 2019). The morphological characteristics between these subspecies are not obvious, which may lead to the confusion of Chinese pangolins of different subspecies through illegal trade. In order to guide rescue and reintroduction for wildlife protection departments as well as for law enforcement, it is necessary to reveal the genetic characteristics of Chinese pangolin.

In order to understand the genetic and evolutionary relationship between Chinese pangolinand and other species, The total DNA in the samples was extracted by using the CTAB method, Sequencing was performed on the Illumina Novaseq platform (Total Genomics Solution Limited, SZHT), The Illumina raw sequence reads were edited using the NGS QC Tool Kit v2.3.3. High-quality reads were assembled into contigs using the de novo assembler SPAdes 3.11.0, Sequence annotation was added using MITOS (http://mitos.bioinf.uni-leipzig.de/index.py). The *Manis pentadactyla aurita* tissue used for DNA extraction was collected from individuals who killed by feral dog in the forest area of Heyuan city, Guangdong province, China (N23°73′, E114°70′). The sample was preserved in the laboratory of Guangdong Academy of Forestry Science, with specimen number 2020-M-Manis-1.

Additionally, we reconstructed the complete mitochondrial genome based on Neighbor-joining tree of the Chinese pangolin and other 7 *Manis* species with Kimura 2-parameter model using MEGA version X (Kumar et al. 2018), the tree was tested with 1 000 Bootstrap replications.

The complete mitogenome of *Manis pentadactyla* is 16 573 bp in length (GenBank accession number MT 335859), which is made up of 13 protein-coding genes, 2 rRNA genes, 22 tRNA genes, and one control region. Except for 1 tRNAs, most mitochondrial genes are encoded on the H-strand as in other mammals. The overall base composition is A 34.5%, C 26.9%, G 13.2%, T 25.4%, with a much higher A + T content.

The phylogenetic Neighbor-joining tree is shown in Figure 1 and bootstrap supports for most of the tree branches are high. From the Neighbor-joining tree, we assumed this study can provide the basis for the genetic evolution of *Manis*.

**Figure 1.**
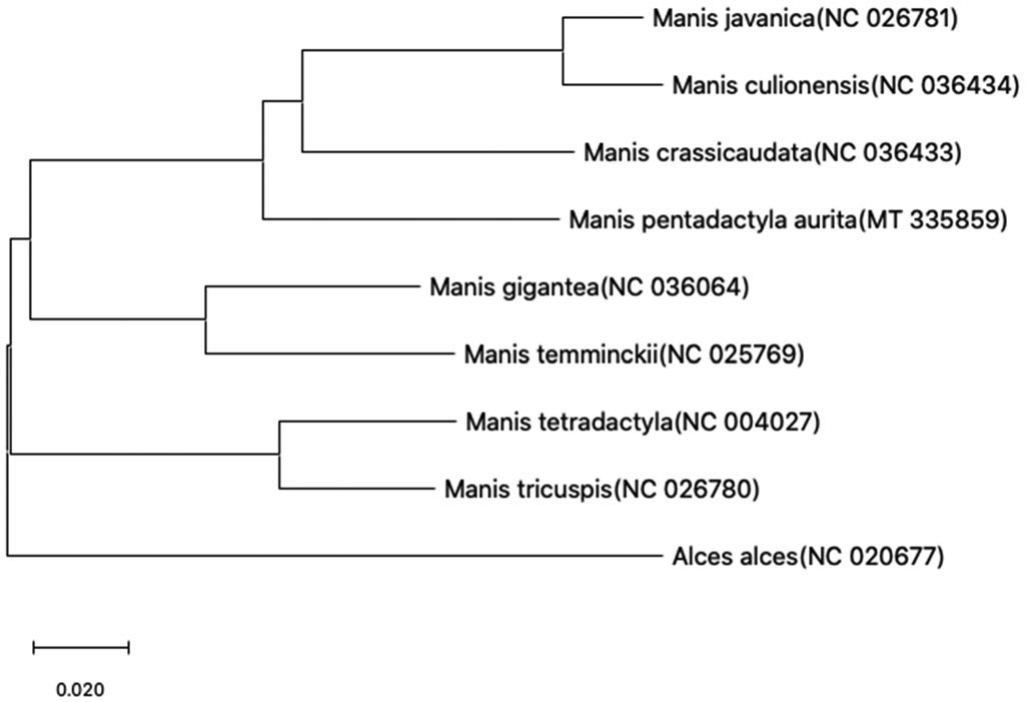
Neighbor-joining phylogenetic tree of *Manis pentadactyla* and other 7 species of *Manis* constructed by MEGA version X. Note: COI (Boykin et al. 2007) of other 7 species of *Manis* are downloaded from NCBI and the GenBank accession numbers are given in the bracket after the species name.

## Data Availability

The data that support the findings of this study are openly available in Genebank at http://www.ncbi.nlm.nih.gov/, reference number MT 335859.
